# Meeting Report: The 8th Barossa Meeting—Cell Signaling in Cancer Medicine in the Barossa Valley, Australia

**DOI:** 10.1038/s41419-018-0285-7

**Published:** 2018-02-15

**Authors:** Guillermo A. Gomez, Joanna M. Woodcock, Vinay Tergaonkar, Philip A. Gregory

**Affiliations:** 1 Centre for Cancer Biology, University of South Australia and SA Pathology, Adelaide, 5000 SA Australia; 2 Institute of Molecular and Cell Biology (IMCB), Singapore, Singapore

The 8th Barossa Meeting in South Australia was organized by the Center for Cancer Biology, around the theme of cell signaling and its application to the development of cancer therapeutics.

## Cancer genomics and epigenetics

Finding new treatments for pediatric cancers forms part of the strategic alliance between Children’s Cancer Institute, the Peter McCallum Cancer Center and the Centre for Cancer Biology. Ali Shilatifard (Northwestern University, USA) opened this meeting and elegantly described the function of a single point mutation in histone H3 that occurs in over 80% of diffuse intrinsic pontine gliomas (DIPG), one of the most devastating forms of pediatric cancers. This histone H3 mutation (H3K27M) was associated with the bromodomain proteins (BRD2/4), which have received considerable attention as therapeutic targets. Treatment of DIPG with the bromodomain and extra-terminal domain (BET) inhibitors significantly reduced tumor progression in mouse models, demonstrating new potential for the treatment of patients affected by this disease.

A greater understanding of the signaling pathways that drive cancer progression is critical for developing new therapeutic targets and strategies. In an engaging talk, Vinay Tergaonkar (Institute of Molecular and Cell Biology, Singapore and Centre for Cancer Biology, Adelaide) described novel non-canonical functions of the telomerase catalytic subunit, TERT, showing that cancer cells respond rapidly to TERT manipulation before effects on telomere length are observed. These activities are mediated by an association of TERT with RNA polymerase III that increases the production of transfer RNAs and concomitant global protein synthesis. Collectively, these findings suggest that combining telomerase inhibitors with drugs that affect translation might represent an efficacious therapeutic strategy for a range of cancers.

Key technological advances in relation to genome editing have revolutionized our ability to elucidate the functions of genetic variants. Lea Starita (University of Washington, USA) adopted saturating CRISPR-Cas9 editing combined with functional screens to prospectively determine pathogenic variants in the BRCA1 gene. These types of approaches will be instrumental in deciphering the functional relevance of variants of unknown significance, which constitute the majority of mutations identified in cancer genomic analyses.

## Cancer cell signaling

**Fig. 1:**
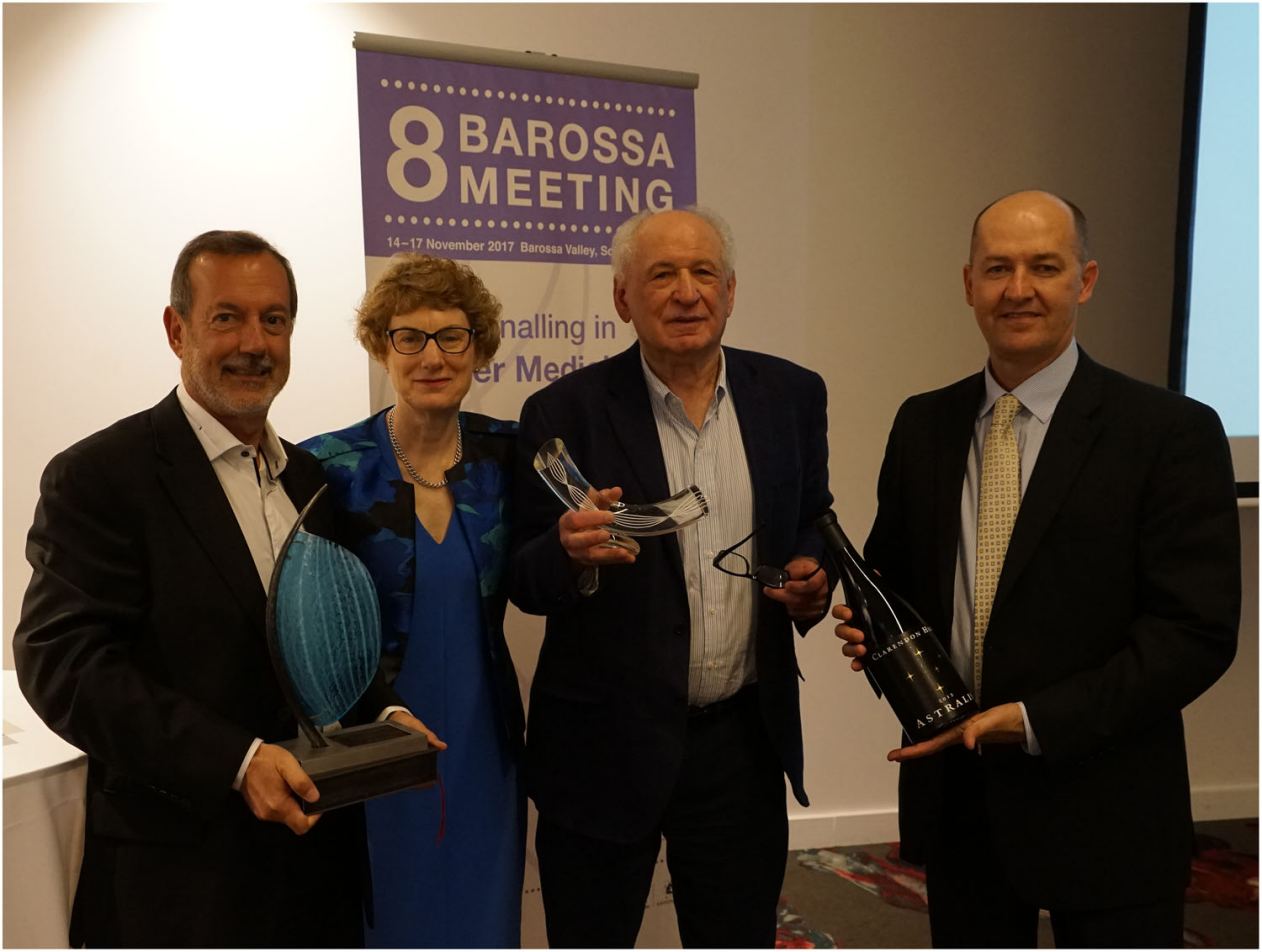
The Clifford Prize for Cancer Research was awarded to Professor Joseph Schlessinger. Left to right: Professor Angel Lopez (Centre for Cancer Biology, Adelaide), Ms Jenny Richter (Central Adelaide Local Health Network), Professor Joseph Schlessinger (Yale University, USA) and Professor Stuart Pitson (Centre for Cancer Biology, Adelaide)

Joseph Schlessinger (Yale University, USA) was the recipient of the Clifford Prize for Cancer Research (Fig. 1) and presented an inspirational lecture about the discovery of receptor tyrosine kinases and their impact on our understanding of both normal and cancer cell signaling. In a pioneering long-term effort, his group has recently identified the structure of the beta-Klotho receptor bound to its primary ligand fibroblast growth factor (FGF) 21, a cytokine that plays key roles in endocrine cell function. This information is being utilized to generate mutant FGF21 ligands with increased binding activity and efficacy for potential use in the treatment of obesity and liver disease.

Recent key advances in understanding the regulation of receptors were discussed. Michael Parker (St Vincent’s Institute of Medical Research and Bio21 Institute, University of Melbourne) presented the highlights of the recently solved crystal structure of the interleukin-3 (IL-3) receptor. This was followed by Winnie Kan (Centre for Cancer Biology, Adelaide), who used this information for her studies using IL-3 receptor mutants to dissect the functional IL-3 receptor signaling complex. These results show how IL-3 receptor complex stoichiometry leads to differential effects on downstream signaling pathways with implications for approaches to target IL-3 signaling for therapy.

## Cancer and stem cell biology

Fiona Watt (Kings College London, UK) described how post-mitotic, differentiated cell layers in the skin contribute to tissue homeostasis and tumor formation. By analyzing mice from the international knockout mouse consortium for epidermal abnormalities, her group applied an unbiased search for important regulators of skin differentiation, identifying keratin 76 (KRT76) as essential for normal skin development and as a key tumor suppressor in several tissues.

Several talks described advances in our understanding of the role of Hippo signaling pathway in cancer progression. Wanjin Hong (Institute of Molecular and Cell Biology, Singapore) described a functional link between the extracellular matrix (ECM) proteoglycan Agrin and co-transcriptional activator YAP. Andrew Cox (Peter McCallum Cancer Center, Melbourne) continued this theme, showing that the YAP-dependent genes are required for metabolic homeostasis during tissue growth. Since upregulation of YAP is associated with a variety of cancers, an understanding of how this pathway is regulated and of its main downstream effector are likely to provide new avenues for the design of anticancer treatments.

## Tumor vascularization/angiogenesis and microenvironment

An important aspect of cancer is the capacity of tumor cells to infiltrate other tissues. During this session, Lena Claeeson-Welsh (Uppsala University, Sweden) and Emma Gordon (Institute for Molecular Biosciences, Brisbane) presented recent findings showing how the VEGF-2-Src signaling axis contributes to the remodeling of endothelial cell–cell junctions required for increased permeability of blood vessels and transmigration of cancerous cells, but also for sprouting of new blood vessels. Inhibiting this process by genetic manipulation of endothelial cells reduces metastasis of tumor cells and the creation of an inflammatory niche that favors tumor metastasis and growth.

It is recognized that the tumor microenvironment plays key roles in cancer progression through many avenues. Maria Sibilia (Medical University of Austria, Vienna) shows that the EGFR plays oncogenic roles when specifically expressed in tumor-associated myeloid cells. EGFR signaling was also shown to be important for the regulation of barrier function and inflammation in the skin, a defect that is normally observed in patients treated with anti-EGFR therapies. Together, it is hoped that this information may aid in improving anti-EGFR based therapies.

## Novel therapies and therapeutic targets

There were several talks that described the development of novel therapies and approaches for cancer treatment. Ross Hannan (Australian National University, Canberra and Peter MaCallum Cancer Centre, Melbourne) described how disruption of ribosome biogenesis, through inhibition of rDNA transcription using the Pol I inhibitor, CX-5461, induces nucleolar stress and is proving to be an effective therapeutic strategy for cancer.

Mutations in the gene RAS are among the most frequently oncogenic events observed in cancer, and are potent drivers of cancer progression. Johannes Bos (University Utrecht, Netherlands) described the development of wild-type and mutant K-RAS colorectal cancer (CRC) organoids in an effort to screen for agents that might inhibit CRC progression. As organoids are genetically diverse and tractable models, they harbor great potential for identifying new potent combinations of drugs for cancer therapy.

In conclusion, the 8th Barossa Meeting showcased many advances in genomic, screening, and model systems in efforts to elucidate the intricacies of signaling pathways that contribute to cancer, and to identify therapies that will be most useful for treating cancers. Moving forward, these studies highlighted the need for utilizing combinations of therapies towards the ultimate goal of personalized medicine.

